# A comparative study reveals a similar validity of telepsychiatry and face-to-face psychiatric assessment in emergency room setting

**DOI:** 10.1192/j.eurpsy.2021.934

**Published:** 2021-08-13

**Authors:** M. Bistre, R. Eitan, O. Linkovsky, I. Barash, A. Juven-Wetzler, G. Katz, Y. Kohn, D. Argo, R. Teplitz, N. Fastovsky, M.-M. Said

**Affiliations:** 1 Research Unit, Jerusalem Mental Health Center, HaRav Rafael Katzenelbogen, Israel; 2 Eitanim Direction, Jerusalem Mental Health Center, HaRav Rafael Katzenelbogen, Israel; 3 Children And Adolescent Unit, Jerusalem Mental Health Center, HaRav Rafael Katzenelbogen, Israel

**Keywords:** telepsychiatry

## Abstract

**Introduction:**

Telepsychiatry (TP) can provide an alternative to traditional face-to-face (FTF) assessments. However, TP in the emergency room setting is much less prevalent, probably due to lack of solid evidence about its effectiveness and acceptability.

**Objectives:**

To directly compare traditional FTF and TP modalities in the emergency room setting.

**Methods:**

Psychiatric patients (n=38) presented to the emergency room went through traditional in-person and videoconference TP interviews in varying order. Both FTF and TP interviewers that examined the patients as well as a third psychiatrist, acting as an observer for both modalities, determined the diagnosis, disposition recommendation and indication for involuntary admission.

**Results:**

Rater decisions had a high matching on disposition and indication for involuntary admission (Cohen’s Kappa (CK) of 0.84/0.81, 0.95/0.87 and 0.89/0.94 for FTF-TP, Observer-FTF and Observer-TP, respectively). Although identical diagnosis matching between the raters was relatively low, the partial diagnosis matching was high (CK of 0.52/0.81, 0.52/0.85 and 0.56/0.85 for FTF-TP, Observer-FTF and Observer-TP, respectively). Telepsychiatry assessments had comparable acceptability in items such as psychiatrists’ certainty and interviewers’ and patients’ satisfaction.

**Conclusions:**

TP and FTF psychiatric assessments in the emergency room settings have similar validity and acceptability. Implementation of TP in emergency room settings might improve the mental health services’ quality and access especially for remote populations. TP is especially important during the COVID-19 pandemic to enable treatment for epidemiologically isolated patients and to protect the medical personnel.

